# Syrian Refugees Seeking Hospital Care in Beirut: A Cross-Sectional Study of Reasons for Hospital Admissions

**DOI:** 10.7759/cureus.42276

**Published:** 2023-07-21

**Authors:** Nour Ibrahim, Marwan Zein, Ali H Abdel Sater, Omar El Khatib, Loubna Tayyara

**Affiliations:** 1 Medicine, Faculty of Medical Sciences, Lebanese University, Beirut, LBN; 2 Pulmonology, Faculty of Medical Sciences, Lebanese University, Beirut, LBN

**Keywords:** united nations, hospital departments, emigrants and immigrants, refugees, delivery of health care

## Abstract

Introduction: Lebanon has the highest Syrian refugee density worldwide. The influx of Syrian refugees has had various impacts on Lebanon, with one of the most significant effects observed in the already exhausted healthcare system. This study aimed to determine the reasons for hospitalization among registered Syrian refugees in Beirut who were admitted to Rafik Hariri University Hospital (RHUH) between December 2017 and June 2020.

Methods: Data from 7,480 diagnosed cases were collected from the RHUH archives between December 2017 and June 2020 and were analyzed using SPSS (IBM Corp., Armonk, NY, USA). The collected data included information related to demographics, admission date, primary diagnosis, and other related medical problems. Variations and correlations were then tested.

Results: Of the cases, 73.4% were females; the mean age was 28 ± 16.23 years. Fifty-seven percent of the admitted cases were solely due to pregnancy, childbirth, and puerperium reasons, and 91.14% of the deliveries were single deliveries by cesarean section. Common reasons for hospitalization were injuries (5.8%) and diseases of the digestive system (6.8%), circulatory system (4.7%), and respiratory system (4.4%). Non-communicable diseases (NCDs) constituted 61% of all hospital admissions, while only 6.6% belonged to communicable diseases. Reasons for hospitalization and the type of diagnosed diseases were associated with gender and age groups (p-values <0.001).

Conclusion: The major reasons for hospitalization among Syrian refugees were related to pregnancies and NCDs. The burden of the Syrian refugee influx on the Lebanese healthcare system can be alleviated by improving community health education, public health services, and conditions for refugees.

## Introduction

The global Syrian refugee crisis

The Syrian crisis began in 2011 and, ever since, Syrian people have been fleeing their country, migrating primarily to neighboring countries as well as European ones [1]. Lebanon, Iraq, Turkey, and Jordan host almost 95% of the overall number of registered Syrian refugees worldwide [2]. Regardless of whether refugees stay in camps or integrate into the host population, they impose health and economic burdens on the healthcare systems and governments of host countries that often struggle to meet their needs [3].

Refugees experience harsh and stressful conditions while leaving their homes and seeking asylum in other countries. They undergo lifestyle changes that place them at a higher risk of developing chronic diseases like cancer, diabetes, and hypertension [4]. In addition to non-communicable diseases (NCDs), Syrian refugees, especially those living in camps, are prone to communicable diseases (CDs) [5]. Together, CDs and NCDs create a double burden on health systems and overall population health [6].

Lebanese healthcare for Syrian refugees

Lebanon has the highest Syrian refugee density worldwide, hosting around 1.5 million Syrian refugees registered with the United Nations High Commission for Refugees (UNHCR), and up to 500,000 unregistered Syrian refugees [7,8]. The majority of the Syrian refugee population resides in the most underserved areas of Lebanon, where many of the existing host population live below the poverty line. This situation has created severe strains and increased burdens on the pre-existing, fragile, and overstretched Lebanese healthcare sector with its finite resources [9].

The Lebanese healthcare system is predominantly private, a major reason why healthcare is expensive and inaccessible for a large proportion of Syrian refugees. Nevertheless, various stakeholders, including the Lebanese Ministry of Public Health (MoPH), UNHCR, and non-governmental organizations (NGOs), have taken steps to improve healthcare availability for Syrian refugees [10]. Syrian refugees seeking healthcare often rely on healthcare centers or services (either run by NGOs or within the Lebanese public system governed by the MoPH) that offer reduced or free care.

In Beirut, the capital of Lebanon, Rafik Hariri University Hospital (RHUH) is considered one of the main public hospitals and the principal public maternity hospital. RHUH sees a high rate of refugee patients, with UNHCR covering up to 75% of their healthcare expenditure for cases strictly related to life-saving emergencies and obstetrical, delivery, and neonatal care. Notably, Doctors Without Borders has operated a midwife-led normal birth center next to RHUH since 2018, referring only complicated cases to RHUH.

The burden of CDs and NCDs among Syrian refugees in Lebanon

In Lebanon, the influx of Syrians has led to major outbreaks like cutaneous leishmaniasis in 2012 and hepatitis A in 2014 [11,12]. Also, the arrival of Syrian refugees in Lebanon has been associated with an increased prevalence of tuberculosis, which had been on a declining trend before 2011 [13]. Additionally, a high prevalence of NCDs (mainly diabetes, hypertension, cardiovascular diseases, and chronic respiratory diseases) has been reported among Syrian refugees residing in Lebanon, leading to an increased demand for hospitalization and healthcare services [14]. Syrian refugees also have a reported higher incidence of birth complications requiring hospitalization and emergency cesarean sections [15].

Several studies have investigated the rate of CDs among Syrian refugees or their prevalence in Lebanon following the influx of Syrian refugees, assessing changes in diseases like tuberculosis [13], cutaneous leishmaniasis [11,16], and hepatitis A virus [12]. Other studies evaluated NCDs like antenatal care [17] and psychiatric disorders [18]. However, these studies are specific to a certain type of disease (CD or NCD) without inter-comparison, and each study was conducted within a different timeframe. Some studies were also based on surveys and literature, without relying on official data from recognized healthcare institutions [2,14].

Although the number of studies assessing CDs and NCDs among Syrian refugees in Lebanon is increasing, there is still a lack of studies combining and comparing the actual reasons for hospitalization among Syrian refugees in Lebanon. Understanding the prevalence rate of each reason for seeking hospital care in a recent, well-defined time interval can help target the most burdensome etiologies by reallocating healthcare expenses, potentially minimizing the overall morbidity and the need for hospital care among the Syrian refugee population.

Hence, this study aims to assess the reasons for seeking hospital care among registered Syrian refugees funded by UNHCR at the RHUH between December 2017 and June 2020.

## Materials and methods

Study design

This was a cross-sectional, single-center study conducted at RHUH, a main hospital in Beirut where refugees are covered by UNHCR, from December 2017 to June 2020. Files of 6,894 Syrian refugee patients, of any gender and age, officially registered in the Lebanese governmental system during the specified timeframe, were retrieved from RHUH’s archived data. Each hospital admission was considered as an entry irrespective of the patient’s identity. As such, 7,480 hospital entries were analyzed in this study to find the reasons for hospitalizations among Syrian refugees in Beirut. Ethical approval was obtained from the institutional review board (IRB) of RHUH.

Syrian refugees’ demographics (age group distributed into ranges of 10 years and gender), admission date (distributed into four periods: March till May, June till August, September till November, December till February of the following year), and primary diagnosis were recorded. Primary diagnosis was also categorized as CD versus NCD and medical disease versus surgical disease. The International Classification of Diseases, 10th Revision, Version 2016 (ICD-10-Version 2016) was followed to assign each primary diagnosis into a certain disease category as follows: A00-B99: Certain infectious and parasitic diseases; C00-D49: Neoplasms; D50-D89: Diseases of the Blood and Blood-Forming Organs and Certain Disorders Involving the Immune Mechanism; E00-E89: Endocrine, Nutritional and Metabolic Diseases; F01-F99: Mental, Behavioral and Neurodevelopmental Disorders; G00-G99: Diseases of the Nervous System; H00-H59: Diseases of the Eye and Adnexa; H60-H95: Diseases of the Ear and Mastoid Process; I00-I99: Diseases of the Circulatory System; J00-J99: Diseases of the Respiratory System; K00-K95: Diseases of the Digestive System; L00-L99: Diseases of the Skin and Subcutaneous Tissue; M00-M99: Diseases of the Musculoskeletal System and Connective Tissue; N00-N99: Diseases of the Genitourinary System; O00-O99: Pregnancy, Childbirth, and the Puerperium; P00-P96: Certain Conditions Originating in the Perinatal Period; Q00-Q99: Congenital Malformations, Deformations, and Chromosomal Abnormalities; R00-R99: Symptoms, Signs, and Abnormal Clinical and Laboratory Findings, Not Elsewhere Classified; S00-T88: Injury, Poisoning, and Certain Other Consequences of External Causes; V00-Y99: External Causes of Morbidity; Z00-Z99: Factors Influencing Health Status and Contact with Health Services; U00-U99: Special purposes.

Statistical analysis

Data was analyzed using SPSS, version 25 (IBM Corp., Armonk, NY, USA). Quantitative variables were denoted as means and standard deviations, whereas categorical variables were denoted as frequencies and percentages. Cross-tabulation was used, followed by the Chi-square test and Cramer’s V coefficient, to find correlations and association strengths between factors such as disease types, diagnoses, departments, age, gender, and date of admission. A P-value < 0.05 was considered as statistically significant for all executed tests. For statistically significant correlations, Cramer’s V effect size was used to interpret the strength of the association: weak or negligible (if ≤ 0.2), moderate (if between 0.2 and 0.6), or strong (if > 0.6).

## Results

In total, our study involved 7,480 hospital inpatient admissions between December 2017 and June 2020. Of the admitted Syrian refugees, 73.4% (5,489) were females and 26.6% (1,991) were males. The mean age of the admitted patients was 28 ± 16.23 years, with patients ranging in age from newborns to 107 years old.

When each primary diagnosis of the hospital admissions was classified based on the International Classification of Diseases (2016), 22 categories were formulated. The most common admitted cases (57%) were found to be related to pregnancy, childbirth and puerperium purposes. This was followed by digestive system diseases (6.8%), injury or poisoning or other consequences of external causes (5.8%), circulatory system diseases (4.7%), respiratory system diseases (4.4%) and genitourinary system diseases (4.1%). Additional, less common causes of hospital admissions were attributed to infectious and parasitic diseases, neoplasms, and mental, behavioral, or neurodevelopmental diseases (Table 1). Out of the total 7,480 hospital admissions, the vast majority [4,572 (61%)] were due to NCDs and only 347 (6.6%) cases were due to CDs. Furthermore, more than half of the hospital admissions (57.8%) targeted the obstetrics and gynecology (OBGYN) department. Otherwise, 23.6% were to the medicine department, 16.8% were to the surgery department, and 1.8% were unassigned.

**Table 1:** The admission diagnoses of Syrian refugees according to ICD-10-Version 2016.

Diagnosis and its code	Frequency (n)	Percent (%)
A00-B99: Certain infectious and parasitic diseases	163	2.2
C00-D49: Neoplasms	95	1.3
D50-D89: Diseases of blood, blood-forming organs, and immune system	270	3.6
E00-E89: Endocrine, nutritional, and metabolic diseases	83	1.1
F01-F99: Mental, behavioral, and neurodevelopmental diseases	16	0.2
G00-G99: Diseases of the nervous system	143	1.9
H00-H59: Diseases of the eye and adnexa	21	0.3
H60-H95: Diseases of the ear and mastoid process	24	0.3
I00-I99: Diseases of the circulatory system	351	4.7
J00-J99: Diseases of the respiratory system	329	4.4
K00-K95: Diseases of the digestive system	509	6.8
L00-L99: Diseases of the skin and subcutaneous tissue	40	0.5
M00-M99: Diseases of the musculoskeletal system and connective tissue	52	0.7
N00-N99: Diseases of the genitourinary system	305	4.1
O00-O99: Pregnancy, childbirth, and puerperium	4264	57
P00-P96: Conditions of perinatal period	106	1.4
Q00-Q99: Congenital malformations, deformations, and chromosomal abnormalities	81	1.1
R00-R99: Not elsewhere classified	93	1.2
S00-T88: Injury, poisoning, and other consequences of external causes	434	5.8
U00-U99: Special purposes	1	0
V00-Y99: External causes	13	0.2
Z00-Z99: Factors influencing health	87	1.2
Total	7480	100

For the correlation between primary diagnosis and gender, results showed that 93 were males and 70 were females when the diagnosed cases were related to infectious and parasitic diseases. Similarly, for injuries and poisoning, there were 334 cases in males and 100 in females. Other diagnosed cases are detailed in Table 2, which also shows a significant association between diagnosis and gender (p-value <0.001). Moreover, of the total 347 cases of CDs among Syrian refugees, 214 were in males and 133 were in females. Contrarily, NCDs were more common in females (2,961) than in males (1,611). Correlation tests revealed a significant, moderate association between gender and type of diagnosed disease (p-value <0.001, R=0.216).

**Table 2:** Cross-tabulation between gender and primary diagnosis of the studied Syrian refugee cases excluding pregnancy, childbirth, and puerperium.

Diagnosis		Gender		Total
		Male	Female	
Infectious and parasitic diseases		93	70	163
Neoplasms		44	51	95
Diseases of blood, blood-forming organs, and immune system		165	105	270
Endocrine, nutritional, and metabolic diseases		38	45	83
Mental, behavioral, and neurodevelopmental diseases		9	7	16
Diseases of nervous system		93	50	143
Diseases of the eye and adnexa		13	8	21
Diseases of ear and mastoid process		18	6	24
Diseases of circulatory system		230	121	351
Diseases of respiratory system		196	133	329
Diseases of digestive system		317	192	509
Diseases of skin and subcutaneous tissue		28	12	40
Diseases of musculoskeletal system and connective tissue		44	8	52
Diseases of genitourinary system		144	161	305
Congenital malformations, deformations, and chromosomal abnormalities		55	26	81
Not elsewhere classified		48	45	93
Injury, poisoning, and other consequences of external causes		334	100	434
Special purposes		1	0	1
External causes		7	6	13
Factors influencing health		53	34	87
Total		1930	1180	3110

Table 3 summarizes the frequency of primary diagnoses across different age groups in the sample population. Among all age groups, certain diseases were more frequently diagnosed in Syrian refugees under 10 years old, such as infectious and parasitic diseases (114 out of 163 cases), neoplasms (23 out of 95 cases), and diseases of the blood and blood-forming organs. It is noteworthy that diseases of the circulatory system were most commonly diagnosed in those aged between 50 and 59 years old, accounting for 101 out of 351 cases. Mental, behavioral, and neurodevelopmental disorders were primarily diagnosed in young adults aged 20 to 49 years, accounting for 14 out of 16 cases. Moreover, individuals aged between 50 and 69 primarily suffered from diseases related to the circulatory system. Cross-tabulation and correlation tests showed a moderate association between the types of diagnosed diseases and age groups, with a p-value of <0.001. The correlation was V=0.223 for diagnosed diseases and V=0.307 for the type of diagnosed disease.

**Table 3:** Distribution of the age groups according to the primary diagnosis of the admitted Syrian refugees excluding pregnancy, childbirth, and puerperium.

Diagnosed Disease	Age Group										Total
	<10	10-19	20-29	30-39	40-49	50-59	60-69	70-79	80-89	>90	
Infectious and parasitic diseases	114	6	12	13	1	3	4	5	4	1	163
Neoplasms	23	8	9	16	10	13	9	6	1	0	95
Diseases of blood, blood-forming organs, and immune system	131	84	17	22	6	5	4	1	0	0	270
Endocrine, nutritional, and metabolic diseases	19	21	15	7	8	5	4	4	0	0	83
Mental, behavioral, and neurodevelopmental diseases	1	0	4	7	3	0	0	1	0	0	16
Diseases of nervous system	44	18	9	13	10	17	19	9	4	0	143
Diseases of the eye and adnexa	11	6	4	0	0	0	0	0	0	0	21
Diseases of ear and mastoid process	12	8	1	2	1	0	0	0	0	0	24
Diseases of circulatory system	15	10	10	25	60	101	80	38	10	2	351
Diseases of respiratory system	175	33	18	33	18	8	19	12	12	1	329
Diseases of digestive system	124	82	96	87	49	25	20	18	7	1	509
Diseases of skin and subcutaneous tissue	11	7	3	7	2	1	7	2	0	0	40
Diseases of musculoskeletal system and connective tissue	8	16	4	5	13	3	2	1	0	0	52
Diseases of genitourinary system	52	32	50	63	41	18	18	19	11	1	305
Congenital malformations, deformations, and chromosomal abnormalities	59	7	9	3	1	0	1	0	1	0	81
Not elsewhere classified	41	10	9	8	4	7	5	8	1	0	93
Injury, poisoning, and other consequences of external causes	127	109	70	54	25	21	14	8	3	3	434
Special purposes	1	0	0	0	0	0	0	0	0	0	1
External causes	5	4	1	1	1	0	0	1	0	0	13
Factors influencing health	31	37	8	7	1	2	0	1	0	0	87
Total	1004	498	349	373	254	229	206	134	54	9	3110

When examining the seasonal distribution of hospital admissions among Syrian refugees, certain diagnoses predominated in specific periods of the year. Throughout the studied years, pregnancy, childbirth, and puerperium were the most common causes of admissions across all seasons. They were followed by diseases of the respiratory system in winter (7.4%) and diseases of the digestive system in spring and summer (8% and 7%, respectively). Both injury, poisoning, and other consequences of external causes and diseases of the digestive system were responsible for 6% of admissions in fall. In terms of overall admissions, the highest rate was observed in winter (28.6%), while the lowest was in the fall season (22%). Table 4 presents the detailed distribution of cases according to diagnosis and date of admission at RHUH. Moreover, results showed that CDs were more common in the cold season from December to February, accounting for 113 out of 347 cases. A similar trend was observed for NCDs, with 713 out of 4,572 cases occurring during the same period. The distribution of each type of diagnosed disease varied throughout the seasons. The results showed a weak association between the type of diagnosed disease and date of admission (p-value <0.001, V =0.054).

**Table 4:** Distribution of the admitted cases pertaining to Syrian refugees depending on the primary diagnosis and date of admission to Rafik Hariri University Hospital (RHUH).

Diagnosis	Date of Admission				Total
	December-February	March-May	June-August	September-November	
Infectious and parasitic diseases	38	46	50	29	163
Neoplasms	26	22	30	17	95
Diseases of blood, blood-forming organs, and immune system	69	93	58	50	270
Endocrine, nutritional, and metabolic diseases	28	20	17	18	83
Mental, behavioral, and neurodevelopmental diseases	3	6	4	3	16
Diseases of nervous system	42	38	31	32	143
Diseases of the eye and adnexa	5	3	7	6	21
Diseases of ear and mastoid process	10	5	3	6	24
Diseases of circulatory system	114	92	79	66	351
Diseases of respiratory system	158	74	39	58	329
Diseases of digestive system	137	153	122	97	509
Diseases of skin and subcutaneous tissue	12	10	7	11	40
Diseases of musculoskeletal system and connective tissue	19	11	5	17	52
Diseases of genitourinary system	97	77	74	57	305
Pregnancy, childbirth, and puerperium	1174	1067	1042	981	4264
Conditions of perinatal period	30	24	24	28	106
Congenital malformations, deformations, and chromosomal abnormalities	18	17	16	30	81
Not elsewhere classified	19	20	28	26	93
Injury, poisoning, and other consequences of external causes	118	120	100	96	434
Special purposes	0	0	0	1	1
External causes	2	1	7	3	13
Factors influencing health	23	28	25	11	87
Total	2142	1927	1768	1643	7480

The majority of hospital admissions among the studied Syrian refugee women for delivery purposes were due to single delivery by cesarean section, accounting for 91.14% of cases. Table 5 provides a detailed breakdown of all diagnosed cases for delivery purposes, which totaled 2,236.

**Table 5:** Frequency and percent distribution of pregnant Syrian refugees in Lebanon according to their mode of delivery.

Delivery related diagnosis	Frequency (n)	Percent (%)
Spontaneous vertex delivery	59	2.64
Spontaneous breech delivery	5	0.22
Other single spontaneous delivery	2	0.09
Single spontaneous delivery, unspecified	3	0.13
Single delivery by forceps and vacuum extractor	2	0.09
Low forceps delivery	2	0.09
Vacuum extractor delivery	2	0.09
Single delivery by caesarean section	2038	91.14
Delivery by elective caesarean section	65	2.91
Delivery by emergency caesarean section	34	1.52
Delivery by caesarean hysterectomy	9	0.4
Other single delivery by caesarean section	1	0.04
Delivery by caesarean section, unspecified	8	0.36
Multiple delivery, all spontaneous	2	0.09
Multiple delivery, all by caesarean section	4	0.18
Total	2236	100

## Discussion

This study is the first to assess the reasons for hospitalization among registered Syrian refugees in Lebanon. In 2014, the International Labor Organization stated that the Syrian refugee population in Lebanon is predominantly young, with almost 62% being under 24 years old [19]. Our study found a similar trend, as it revealed a preponderance of a younger age group within the sampled Syrian refugees. Reports indicate that 57-59% of hospital admissions among Syrian refugees are related to pregnancy [20], which aligns with our findings. The high rate of pregnancies and childbirth complications among Syrian refugees in Lebanon can be attributed to the lack or limited use of contraceptives, as well as insufficient access to antenatal care. Both are impeded by various barriers including prohibitive costs, unavailability, religious and cultural considerations, as well as inadequate awareness and knowledge [21]. Furthermore, an alarming percentage (91.14%) of delivery cases involved hospital admissions for single deliveries by cesarean section, which is substantially higher than the cesarean section estimates among Lebanese women (49%) [22]. This contradicts a study by Huster et al., which reported that out of the 6,366 deliveries involving Syrian refugees in Lebanon, only 35% were by cesarean section [15]. One possible explanation for this high rate of cesarean sections could be that the majority of pregnant Syrian refugees rarely visit a healthcare provider during their pregnancy. This can lead to an increased likelihood of complications diagnosed at the time of delivery, necessitating emergency cesarean sections, such as in cases of pre-eclampsia, placenta previa, and eclampsia [21]. In addition, the lack of supervision, absence of national guidelines for standards of care, and possible abuse by obstetricians - who may opt for cesarean sections for increased reimbursement - may also contribute to these observed high rates among refugees. For instance, it's estimated that the average costs of cesarean deliveries, including prenatal care, childbirth, and postnatal care, are 15-30% higher than vaginal deliveries [23]. Furthermore, the high rate of cesarean sections may also be due to selection bias, as the studied sample only included those who presented to RHUH, rather than encompassing all Syrian refugee deliveries. Generally, Lebanon seems to encourage delivery through cesarean section, as confirmed by a study conducted in 2007, despite it being associated with poorer maternal and fetal outcomes and leading to inappropriate use of medical resources [24]. All of this underscores the need for increased awareness among Syrian refugees residing in Lebanon regarding modes of delivery, antenatal care, contraception, and family planning. There is also an immense need for the implementation of strategies or new health policies to reduce the number of unnecessary and unindicated cesarean deliveries in Lebanon.

Other common reasons for hospitalization among the surveyed Syrian refugees included diseases of the digestive system (6.8%), injuries, poisoning, or other consequences of external causes (5.8%), diseases of the circulatory system (4.7%), and diseases of the respiratory system (4.4%). Doocy et al. similarly reported that the primary reasons for hospitalization among Syrian refugees in Jordan were injuries (20.7%), cardiovascular diseases (13.7%), respiratory diseases (12.2%), and conditions of the digestive system (7%) [25]. In Turkey, the most common diagnoses among Syrian refugees were respiratory diseases (14.4%), diseases of the eye and adnexa (12.6%), and cases of injuries, poisoning, or other consequences of external causes (10.7%) [26]. However, it's notable that in our study, only 0.3% of hospital admissions were due to diseases of the eye and adnexa. Injuries often feature prominently as a reason for seeking hospital care, likely owing to the harsh living conditions and informal work environments that the refugees have to endure [25]. Furthermore, war-related injuries are quite common in Lebanon due to its proximity to the Syrian border [2]. During the study period, only 16 (0.2%) hospital admissions were related to mental, behavioral, or neurodevelopmental disorders. Conversely, symptoms of mental disorders and illnesses were quite prevalent among Syrian refugees migrating to Switzerland, affecting nearly 47% of individuals [27]. In Greece, Farhat et al. found that more than 75% of the 1,293 refugees studied were diagnosed with an anxiety disorder [28]. The discrepancy between our results and those of the aforementioned studies could be due to a hesitancy in seeking mental health care among the refugee population. This reluctance might stem from religious considerations, a lack of understanding of mental disorders, cost barriers, and stigma - factors that also affect the general Lebanese population's access to mental health care [29].

There has been a general shift from CDs to NCDs among both the Syrian and Lebanese populations. Reports indicate a dramatic increase in NCD cases among Syrians, especially following the destruction of major hospitals and the ensuing negative impact on prevention and treatment of many chronic diseases [14]. Factors contributing to this rise in NCDs include the stress of displacement, changes in activity levels and diet, lifestyle changes, and discontinuation of treatment for existing chronic diseases [4,18]. Indeed, Torun et al. found that chronic diseases were very common among Syrian refugees in Istanbul, with at least one member having a chronic disease in three out of four households [30]. However, rates of CDs and NCDs were almost equal among Syrian refugees in Jordan (21.5% versus 21.1%) [25]. The discrepancy in CD versus NCD rates between our study and others may be attributed to variations in how diseases are classified within each diagnostic category. Regardless, screening and preventive measures to limit the spread of CDs are essential steps to preserving the health of all residents in Lebanon, including refugees and national citizens, and to preventing potential outbreaks.

Moreover, the nature of the primary diagnoses appears to be influenced by age. Many diagnosed diseases were reported primarily among those less than 10 years old, compared to their counterparts in older age groups. Mental health illnesses were strictly diagnosed in young adults. Doocy et al. reported that the most common health issues among Syrian refugee women were related to pregnancy and antenatal care [14]. Indeed, Syrian refugee women and young children were particularly vulnerable, as they are prone to family abuse, exploitation, early marriage, malnutrition, and poor hygiene conditions, thus exposing them to high risks of respiratory, dermatological, and gastrointestinal conditions [5,17]. Incidents of injuries and poisoning were expected to be higher in males, especially those working in agricultural jobs and construction sites where they're exposed to dangerous chemicals and accidents, or in low-wage positions associated with hazards [25].

The limitations of the study include its basis on single-center data and the inability to encompass unregistered Syrian refugees. Additionally, RHUH, due to its specialized programs and association with UNHCR, receives the majority of refugees, which could potentially lead to selection bias.

## Conclusions

Awareness campaigns are needed to enhance the knowledge of Syrian refugee patients regarding various health conditions and to promote appropriate practices for screening, treatment, and prevention of these diseases, whether they are communicable or non-communicable. Improving public health services and conditions for refugees could reduce healthcare utilization. Extensive education on modes of delivery, antenatal care, reproductive needs, birth control methods, and family planning should be widely disseminated. Also, in order to limit the re-emergence of many infectious diseases, healthcare strategies should be optimized to increase vaccination coverage, thereby reducing transmission among Lebanese residents. Such efforts are crucial to alleviate the burden imposed by the influx of Syrian refugees on Lebanon's already strained healthcare system. Therefore, future studies addressing the causes of burden on the Lebanese healthcare system and implementing strategies for its recovery are of paramount importance.
